# Hyperglycaemia‐related complications at the time of diagnosis can cause permanent neurological disability in children with neonatal diabetes

**DOI:** 10.1111/dme.13328

**Published:** 2017-06-18

**Authors:** J. O. Day, S. E. Flanagan, M. H. Shepherd, A. W. Patrick, N. Abid, L. Torrens, A. J. Zeman, K. A. Patel, A. T. Hattersley

**Affiliations:** ^1^ Royal Devon and Exeter NHS Foundation TrustExeterUK; ^2^ Institute of Biomedical and Clinical ScienceUniversity of Exeter Medical SchoolExeterUK; ^3^ National Institute for Health Research (NIHR) Exeter Clinical Research FacilityExeterUK; ^4^ Edinburgh Centre for Endocrinology and DiabetesNHS LothianEdinburghUK; ^5^ Royal Belfast Hospital for Sick ChildrenBelfastUK; ^6^ Neuroport LtdDidcotUK; ^7^ Cognitive and Behavioural NeurologyUniversity of Exeter Medical SchoolExeterUK

## Abstract

**Background:**

Children with neonatal diabetes often present with diabetic ketoacidosis and hence are at risk of cerebral oedema and subsequent long‐term neurological deficits. These complications are difficult to identify because neurological features can also occur as a result of the specific genetic aetiology causing neonatal diabetes.

**Case reports:**

We report two cases of neonatal diabetes where ketoacidosis‐related cerebral oedema was the major cause of their permanent neurological disability. Case 1 (male, 18 years, compound heterozygous *ABCC8* mutation) and case 2 (female, 29 years, heterozygous *KCNJ11* mutation) presented with severe diabetic ketoacidosis at 6 and 16 weeks of age. Both had reduced consciousness, seizures and required intensive care for cerebral oedema. They subsequently developed spastic tetraplegia. Neurological examination in adulthood confirmed spastic tetraplegia and severe disability. Case 1 is wheelchair‐bound and needs assistance for transfers, washing and dressing, whereas case 2 requires institutional care for all activities of daily living. Both cases have first‐degree relatives with the same mutation with diabetes, who did not have ketoacidosis at diagnosis and do not have neurological disability.

**Discussion:**

Ketoacidosis‐related cerebral oedema at diagnosis in neonatal diabetes can cause long‐term severe neurological disability. This will give additional neurological features to those directly caused by the genetic aetiology of the neonatal diabetes. Our cases highlight the need for increased awareness of neonatal diabetes and earlier and better initial treatment of the severe hyperglycaemia and ketoacidosis often seen at diagnosis of these children.

## What's new?

- The cases in this report show that children with neonatal diabetes can sustain irreversible neurological damage as a result of complications of ketoacidosis at diagnosis and not just because of the neurological phenotype caused by genetic mutations.
- Both cases presented with diabetic ketoacidosis, required intensive care and probably developed cerebral oedema, leading to severe permanent spastic tetraplegia.
- The long‐term neurological disability means they need intensive institutional or home support and so has a profound impact on their lives.
- Our cases highlight the need for increased awareness of neonatal diabetes and earlier and better initial treatment of the severe hyperglycaemia and ketoacidosis often seen at diagnosis of these children.

**What's new?**

- The cases in this report show that children with neonatal diabetes can sustain irreversible neurological damage as a result of complications of ketoacidosis at diagnosis and not just because of the neurological phenotype caused by genetic mutations.
- Both cases presented with diabetic ketoacidosis, required intensive care and probably developed cerebral oedema, leading to severe permanent spastic tetraplegia.
- The long‐term neurological disability means they need intensive institutional or home support and so has a profound impact on their lives.
- Our cases highlight the need for increased awareness of neonatal diabetes and earlier and better initial treatment of the severe hyperglycaemia and ketoacidosis often seen at diagnosis of these children.

## Introduction

The main cause of morbidity and mortality in childhood‐onset diabetes is ketoacidosis 1. The most common serious complication of diabetic ketoacidosis is cerebral oedema, which carries significant risk of death (21–24%) and permanent neurological disability (18–35% of survivors) 2, 3, 4. Younger children, especially those aged <2 years, and children with delayed diagnosis are more likely to present with diabetic ketoacidosis 5, 6, 7.

Ketoacidosis is common in children with neonatal diabetes who present before 6 months of age 8. The diagnosis is often delayed because osmotic symptoms are difficult to detect in infants. The management of diabetic ketoacidosis is extremely challenging in infants because of the difficulties in managing fluid balance. This increases their risk of cerebral oedema which can cause permanent neurological deficit 9.

It is difficult to determine the contribution of diabetic ketoacidosis‐related complications, such as cerebral oedema, at the time of diagnosis towards overall neurological morbidity in people with neonatal diabetes. Nine of the 22 genetic aetiologies of neonatal diabetes including the most common, *KCNJ11,* may have neurological features attributable to the underlying mutation 10; therefore, neurological features in these people may be assumed to be caused by the underlying genetic aetiology rather than complications at time of diagnosis. We report two cases with neonatal diabetes in whom ketoacidosis‐related cerebral oedema was the major cause of their permanent neurological disability rather than the underlying genetic aetiology.

## Case identification and assessment

### Case identification

Our cases were identified from 125 people with neonatal diabetes (diabetes diagnosed at age <6 months, gestational age >32 weeks) referred from the UK to Exeter Molecular Genetic laboratory and confirmed to have monogenic aetiology for their diabetes before December 2015 (Table S1). There were three cases in whom persistent neurological features were severe and unexpected for the given mutation when compared with others in the cohort with the same mutation or mutations in the same gene and/or compared with the published literature. Initial identification of these cases was from the clinical information provided by the referring physicians based on patients’ health records and subsequently validated on clinical examination. The clinical details at the time of diagnosis of diabetes were reviewed from the hospital clinical records. This was available for two cases but not for the third case as the early clinical notes had been destroyed. The third case is therefore not reported.

### Neurological and functional assessment

We performed detailed neurological examination on these cases and assessed their disability using the WHO Disability Assessment Schedule 2.0 (WHODAS 2.0) 11. Scores in six different domains are calculated to give values 0 (no disability in daily living) to 100 (severe disability) and can be completed by carers or family members. Family members with the same mutation who did not present with severe diabetic ketoacidosis were assessed as controls for comparison.

### Ethics

This study was given ethical approval by the North Wales Research Ethics Committee, UK.

## Case 1

### Early history

**Figure 1:**
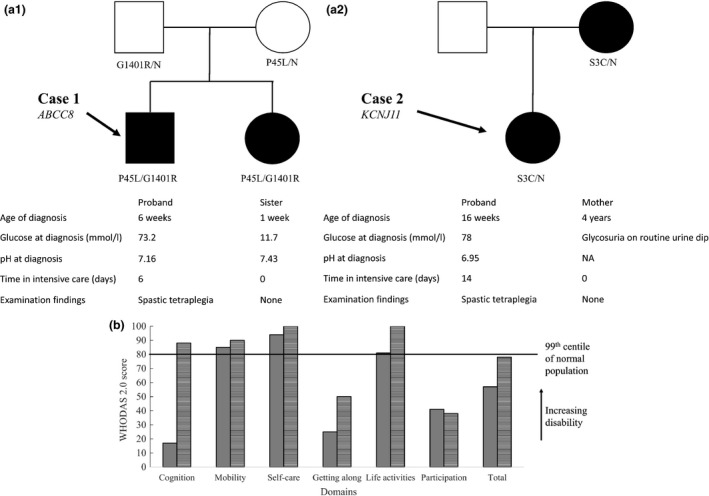
(a) Pedigrees of the cases. Information is provided on their mutation status, clinical features at diabetes presentation and their present neurological condition. Filled symbols are those with diabetes. (b) WHO Disability Assessment Schedule 2.0 (WHODAS 2.0) quality‐of‐life questionnaire. Both cases showed high levels of overall disability with most domain scores > 99th centile for the normal population. Case 1 solid grey, case 2 horizontal lines.

Case 1 was born at term and had increased hunger and frequent wet nappies from 3 weeks of age (Fig. 1a). He was diagnosed with diabetes at 6 weeks when he presented with severe dehydration and diabetic ketoacidosis (pH 7.16, blood glucose 73.2 mmol/l). He had reduced consciousness, opisthotonus and partial seizures and subsequently aspirated. He was diagnosed as having cerebral oedema, required intensive care for 6 days and was hospitalized for 25 days. At 6 months he had motor developmental delay, bilateral spasticity and elevated muscle tone. A genetic diagnosis of a compound heterozygous *ABCC8* p.P45L/p.G1401R mutation was made at the age of 8 years and he has been successfully treated with glibenclamide since then. He currently takes glibenclamide 5 mg twice a day and has an HbA_1c_ of 34 mmol/mol (5.3%).

### Current functional status

He is now 18 years old and attends a school for the physically disabled. He uses an electric wheelchair to mobilize and needs assistance for transfers, washing and dressing. On examination, there was marked weakness, increased tone with spasticity, brisk reflexes and contractures of all limbs. His language skills have developed normally. His total WHODAS 2.0 score is 57 (96th centile; Fig. 1b).

### Family member control

The boy's younger sister with diabetes has the same genetic mutation but was diagnosed by capillary blood glucose testing by her parents at 7 days of age before she had symptoms or diabetic ketoacidosis. She has normal development milestones and neurological examination results were normal (Fig. 1a).

## Case 2

### Early history

Case 2 was born at term. She had polyuria and thrush at 16 weeks of age (Fig. 1a). She was diagnosed with diabetes when she presented with reduced consciousness, generalized tonic‐clonic seizures and diabetic ketoacidosis (pH 6.95, glucose 78 mmol/l). She was clinically treated for cerebral oedema and had recurrent seizures requiring intensive care for 2 weeks. One month after discharge she developed spastic tetraplegia (Fig. 1a). A genetic diagnosis of a heterozygous *KCNJ11* p.S3C mutation was made at the age of 19 years. She was transferred from insulin to glibenclamide at the age of 27 years and currently takes glibenclamide 35 mg twice a day and has an HbA_1c_ of 69 mmol/mol (8.5%).

### Current functional status

She is now aged 29 years and lives in residential care. She requires assistance with all activities of daily living. She can say single words and nod her head for communication. Examination revealed spasticity and contractures of all limbs. Her total WHODAS 2.0 score is 78 (99th centile; Fig. 1b).

### Family member control

The girl's mother was diagnosed with diabetes aged 4 years after the detection of glycosuria on a routine urine test without diabetic ketoacidosis. The mother's genetic diagnosis was established at 38 years of age after her daughter's diagnosis. She has normal developmental milestones and examination results were normal (Fig. 1a).

## Discussion

These cases highlight that neurological disability in neonatal diabetes can occur as a result of diabetic ketoacidosis‐related complications at diagnosis in addition to being a direct result of underlying genetic aetiology.

Underlying genetic aetiology is not the major cause of the neurological disabilities seen in these cases. Both cases were extremely unwell at diagnosis with cerebral oedema, as shown by their biochemistry, clinical presentation and the need for intensive care. The family members with the same mutation but without diabetic ketoacidosis/cerebral oedema at presentation did not show any permanent neurological deficit. There were no other cases with *KCNJ11* (*n *=* *44, excluding the third case that we have not reported here) and *ABCC8* (*n *=* *16) neonatal diabetes who had similar neurological deficits in our cohort. Children with *KCNJ11/ABCC8* neonatal diabetes commonly have hypotonia and coordination difficulties but not the spasticity and increased tone in all four limbs seen in our cases 12.

Cerebral oedema related to diabetic ketoacidosis is the likely mechanism for the neurological deficits in these cases. It is known that cerebral oedema attributable to diabetic ketoacidosis leads to permanent neurological features, particularly motor disability, in up to 35% of surviving children 2, 3, 4, 13, 14. Both cases fit the clinical criteria for cerebral oedema 15 and spastic tetraplegia can result from diabetic ketoacidosis with cerebral oedema or other intracranial complications of diabetic ketoacidosis 13, 14, 16, 17, 18. A case with *KCNJ11* neonatal diabetes and neurological deficits attributable to cerebral oedema has been previously reported 9. Although we do not have brain imaging studies for the cases at the time of diabetic ketoacidosis supporting the presence of cerebral oedema, this is not required to make a diagnosis, especially as 40% of cases with clinical cerebral oedema do not show acute abnormalities on their initial computed tomography examinations 15; therefore, it has been proposed that cerebral oedema in the presence of diabetic ketoacidosis is a clinical, not a radiological, diagnosis 15. The severity of disability in these cases may be attributable to late recognition of symptoms leading to severe ketoacidosis, the difficulty of neonatal fluid management and the vulnerability of the developing brain.

These two cases show that not all neurological deficits in people with neonatal diabetes are genetic in origin**.** This is particularly relevant to genetic aetiologies where there is a variable phenotype, such as *KCNJ11* and *ABCC8* neonatal diabetes in which ~20% of people have marked neurological features 8; therefore, neurological features attributed to the genetic change may not be the correct explanation for a person's condition. This information is also important for clinicians when communicating the diagnosis to patient and family members, particularly the likelihood of developing neurological disability in other family members who have inherited the same mutation.

In conclusion, long‐term neurological morbidity in people with neonatal diabetes can occur independently of the underlying genetic mutation because of severe ketoacidosis‐related complications at the time of diagnosis. Our cases highlight the need for increased awareness of neonatal diabetes and earlier and better initial treatment of the severe hyperglycaemia and ketoacidosis often seen at diagnosis of these children.

## Funding sources

M.H.S. is employed as a core member of the National Institute for Health Research (NIHR) funded Exeter Clinical Research Facility. A.T.H. is an NIHR Senior Investigator and a Wellcome Trust Senior Investigator (WT098395/Z/12/Z), K.A.P. has a Wellcome Trust Postdoctoral Training Fellowship (110082/Z/15/Z) and S.E.F. has a Sir Henry Dale Fellowship, jointly funded by the Wellcome Trust and the Royal Society (105636/Z/14/Z). This article presents independent research funded by Wellcome Trust supported by the NIHR Exeter Clinical Research Facility. The views expressed are those of the author(s) and not necessarily those of the NIHR or the Department of Health.

## Competing interests

None declared.

## Supporting information


**Table S1.** Clinical characteristics and genetic aetiology of all 125 patients.
