# Supplementary material for: Hyperglycaemia‐related complications at the time of diagnosis can cause permanent neurological disability in children with neonatal diabetes
Source: Diabet Med. 2017 Jun 18;34(7):1000–4. doi: 10.1111/dme.13328 (PMC5488205; doi:10.1111/dme.13328)
Supplement: Supplementary file 1 — Table S1. Clinical characteristics and genetic aetiology of all 125 patients. [file DME-34-1000-s001.docx]

**Supplementary table 1. Clinical characteristics and genetic aetiology of all 125 patients**

| Age at diagnosis of diabetes (weeks), median (IQR), n | 4 (1-10), n=125 |
| --- | --- |
| Age at referral for genetic testing (weeks), median (IQR), n | 102 (7-880), n=124 |
| Female, n (%), n | 63 (50), n=125 |
| Birthweight (sds), median (IQR), n | ^-^1.97 (^-^2.79-^-^1.12), n=100 |
| Glucose at presentation (mmol/l), median (IQR), n | 27 (20- 41), n= 85 |
| Genetic aetiology, n |  |
| *KCNJ11* | 46 |
| *ABCC8* | 17 |
| *6q24* | 17 |
| *INS* | 16 |
| *GCK* | 7 |
| *GATA6* | 6 |
| *GLIS3* | 4 |
| *EIF2AK3* | 3 |
| *FOXP3* | 3 |
| *PTF1A* | 1 |
| *SLC19A2* | 1 |
| *ZFP57* | 1 |
| *COQ9* | 1 |
| *GATA4* | 1 |
| *NEUROD1* | 1 |
